# P-1926. Clinical Characteristics and 90-Day Mortality in Adults with Cryptococcal Meningoencephalitis

**DOI:** 10.1093/ofid/ofaf695.2095

**Published:** 2026-01-11

**Authors:** Bayard A Taylor, Ameera Jamshad, Daniel B Chastain, Charlie A Garcia, Andrés F Henao Martínez

**Affiliations:** University of Georgia, Ellerslie, GA; The University of Georgia College of Pharmacy, Marietta, GA; University of Georgia College of Pharmacy, Albany, GA; Colorado School of Public Health Anschutz, Aurora, Colorado; University of Colorado Anschutz Medical Campus, Aurora, Colorado

## Abstract

**Background:**

Cryptococcal meningoencephalitis (CM) is a life-threatening opportunistic infection, particularly among immunocompromised individuals. Identifying clinical characteristics and risk factors associated with mortality may improve risk stratification and guide management to optimize outcomes. This study aimed to describe adults diagnosed with CM and compare characteristics between survivors and non-survivors.

**Figure:**
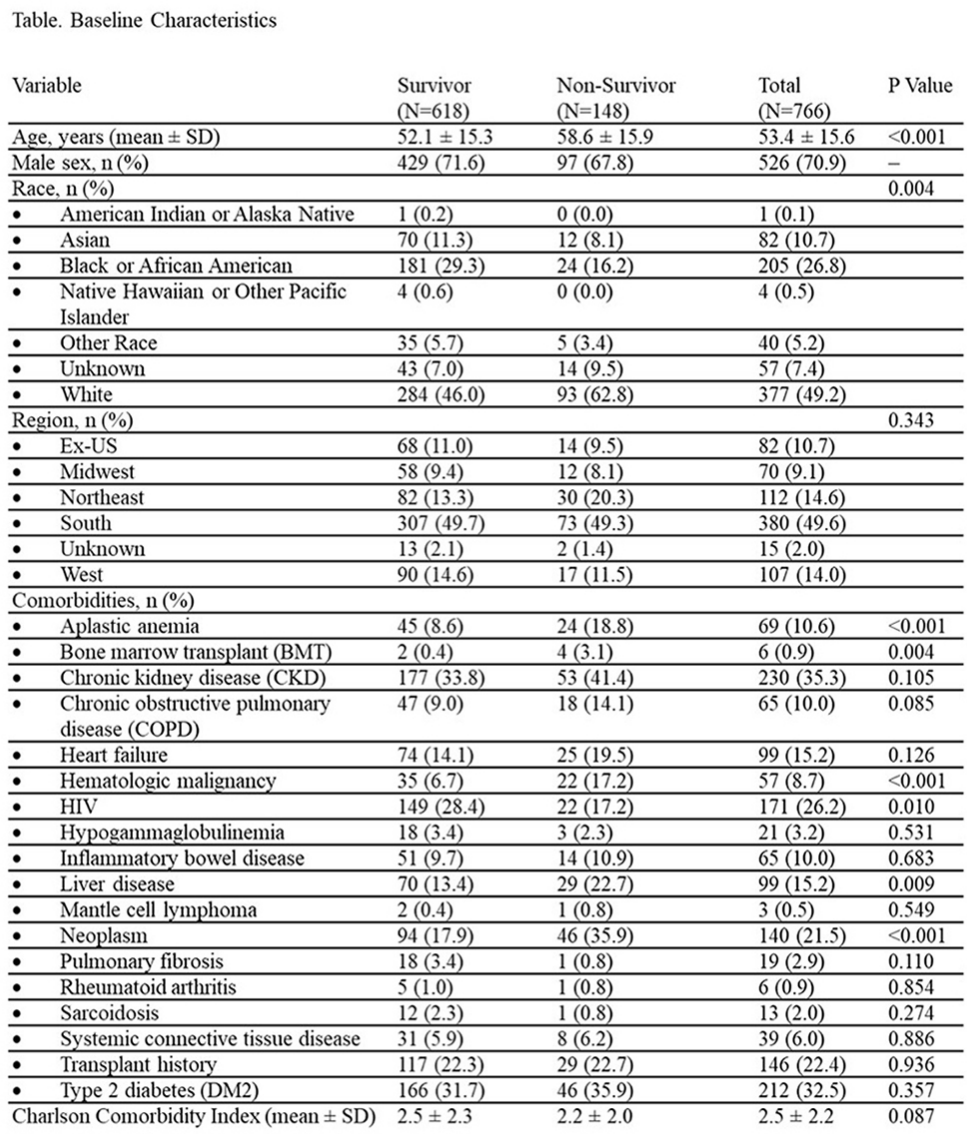

**Figure:**
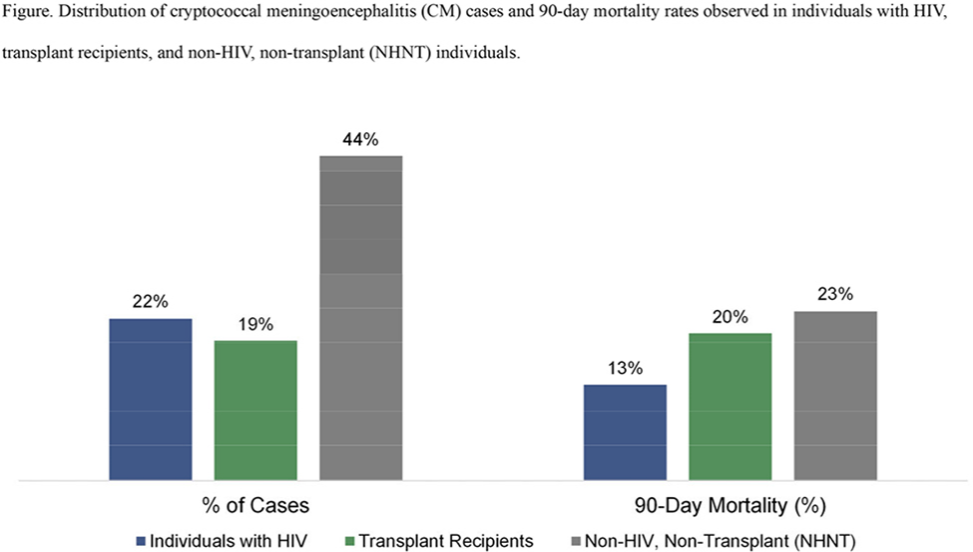

**Methods:**

We conducted a retrospective cohort study using data from TriNetX, a global federated health research network. Adults (≥ 18 years) diagnosed with CM—defined by an ICD-10-CM code for cerebral cryptococcosis or laboratory evidence of cryptococcal antigen or DNA in cerebrospinal fluid—between 2003 and 2024 were included. To enhance diagnostic specificity, all patients were required to have received amphotericin B. Demographics, comorbidities, use of immunosuppressive therapies, and 90-day mortality were assessed and compared between survivors and non-survivors.

**Results:**

Among 766 patients with CM, 26% had HIV, 22% had a history of solid organ transplantation, and 52% had neither condition (non-HIV, non-transplant [NHNT] group) (Figure). Overall, 90-day mortality was 19%, with the highest mortality observed in the NHNT group (23%), followed by transplant recipients (20%) and patients with HIV (13%). Non-survivors were significantly older than survivors (mean age 59 vs 52 years, p< .001) and more likely to identify as White (63% vs 46%) and less likely as Black or African American (16% vs 29%) (p=.004) (Table). Comorbid conditions more frequently observed among non-survivors included neoplasms (36% vs 18%, p< .001), liver disease (23% vs 13%, p=.009), hematologic malignancies (17% vs 7%, p< .001), aplastic disorders (19% vs 9%, p< .001), and prior bone marrow transplant (3% vs 0.4%, p=.004).

**Conclusion:**

NHNT individuals accounted for the majority of cases and experienced the highest mortality. Conversely, HIV-associated CM was more common among survivors, suggesting that advances in HIV-related CM recognition and management may have improved outcomes. These findings reflect a shift in CM epidemiology, with a growing burden among NHNT patients who often have complex comorbidities and immunosuppressive exposures.

**Disclosures:**

Andrés F. Henao Martínez, MD, MPH, F2: Grant/Research Support|Scynexis: Grant/Research Support

